# The impact of dietary risk factors on the burden of non-communicable diseases in Ethiopia: findings from the Global Burden of Disease study 2013

**DOI:** 10.1186/s12966-016-0447-x

**Published:** 2016-12-16

**Authors:** Yohannes Adama Melaku, Awoke Misganaw Temesgen, Amare Deribew, Gizachew Assefa Tessema, Kebede Deribe, Berhe W. Sahle, Semaw Ferede Abera, Tolesa Bekele, Ferew Lemma, Azmeraw T. Amare, Oumer Seid, Kedir Endris, Abiy Hiruye, Amare Worku, Robert Adams, Anne W. Taylor, Tiffany K. Gill, Zumin Shi, Ashkan Afshin, Mohammad H. Forouzanfar

**Affiliations:** 1 School of Public Health, Mekelle University, Mekelle, Ethiopia; 2 Population Research and Outcome Studies, School of Medicine, The University of Adelaide, Adelaide, SA Australia; 3 Institute of Health Metrics and Evaluation, University of Washington, Seattle, USA; 4 KEMRI-Wellcome Trust Research Programme, Kilifi, Kenya; 5 Nuffield Department of Clinical Medicine, University of Oxford, Oxford, UK; 6 St. Paul Millennium Medical College, Addis Ababa, Ethiopia; 7 Department of Reproductive Health, University of Gondar, Gondar, Ethiopia; 8 School of Public Health, The University of Adelaide, Adelaide, Australia; 9 Brighton & Sussex Medical School, Brighton, UK; 10 School of Public Health, Addis Ababa University, Addis Ababa, Ethiopia; 11 Federal Ministry of Health, Addis Ababa, Ethiopia; 12 Centre of Cardiovascular Research & Education in Therapeutics, Department of Epidemiology and Preventive Medicine, Monash University, Melbourne, VIC Australia; 13 Institute of Biological Chemistry and Nutrition, Hohenheim University, Stuttgart, Germany; 14 Department of Public Health, Madda Walabu University, Bale Goba, Ethiopia; 15 Discipline of Psychiatry, School of Medicine, The University of Adelaide, Adelaide, Australia; 16 School of Medicine and Health Sciences, Bahir dar University, Bahir Dar, Ethiopia; 17 Department of Epidemiology, University Medical Center Groningen, the University of Groningen, Groningen, The Netherlands; 18 Department of Public Health, Addis Continental Institute of Public Health, Addis Ababa, Ethiopia; 19 Health observatory, Discipline of Medicine, The Queen Elizabeth Hospital Campus, The University of Adelaide, Adelaide, Australia

**Keywords:** Dietary risks, Non-communicable diseases, Burden of disease, Ethiopia

## Abstract

**Background:**

The burden of non-communicable diseases (NCDs) has increased in sub-Saharan countries, including Ethiopia. The contribution of dietary behaviours to the NCD burden in Ethiopia has not been evaluated. This study, therefore, aimed to assess diet-related burden of disease in Ethiopia between 1990 and 2013.

**Method:**

We used the 2013 Global Burden of Disease (GBD) data to estimate deaths, years of life lost (YLLs) and disability-adjusted life years (DALYs) related to eight food types, five nutrients and fibre intake. Dietary exposure was estimated using a Bayesian hierarchical meta-regression. The effect size of each diet-disease pair was obtained based on meta-analyses of prospective observational studies and randomized controlled trials. A comparative risk assessment approach was used to quantify the proportion of NCD burden associated with dietary risk factors.

**Results:**

In 2013, dietary factors were responsible for 60,402 deaths (95% Uncertainty Interval [UI]: 44,943-74,898) in Ethiopia—almost a quarter (23.0%) of all NCD deaths. Nearly nine in every ten diet-related deaths (88.0%) were from cardiovascular diseases (CVD) and 44.0% of all CVD deaths were related to poor diet. Suboptimal diet accounted for 1,353,407 DALYs (95% UI: 1,010,433-1,672,828) and 1,291,703 YLLs (95% UI: 961,915-1,599,985). Low intake of fruits and vegetables and high intake of sodium were the most important dietary factors. The proportion of NCD deaths associated with low fruit consumption slightly increased (11.3% in 1990 and 11.9% in 2013). In these years, the rate of burden of disease related to poor diet slightly decreased; however, their contribution to NCDs remained stable.

**Conclusions:**

Dietary behaviour contributes significantly to the NCD burden in Ethiopia. Intakes of diet low in fruits and vegetables and high in sodium are the leading dietary risks. To effectively mitigate the oncoming NCD burden in Ethiopia, multisectoral interventions are required; and nutrition policies and dietary guidelines should be developed.

**Electronic supplementary material:**

The online version of this article (doi:10.1186/s12966-016-0447-x) contains supplementary material, which is available to authorized users.

## Background

The burden of non-communicable diseases (NCDs) in sub-Saharan Africa has increased significantly over the past two decades [1–3]. The number of NCD deaths in the region has risen by 68.0% between 1990 and 2013 [4]. In Ethiopia, despite the prevailing high burden of communicable diseases, the proportion of deaths due to NCDs has increased by 73.7% in these years [1–3, 5] and in 2013, more than a third (35.1%) of all deaths were caused by NCDs. CVD was the second most common causes of death behind specific infectious diseases (diarrhoea, lower respiratory and other infectious diseases together), accounting for 121,211 deaths (16.2% of all deaths) in the country [3]. Neoplasms were the fifth most common causes of deaths accounting for 45,520 deaths (6.1% of all deaths) [3, 4].

NCDs are predisposed by various risk factors including behavioural, environmental and metabolic. There is increasing and strong evidence of a causal link between dietary behaviours and patterns, nutrients and NCDs [6, 7]. In sub-Sahara Africa, in addition to problems of undernutrition, dietary factors were responsible for 5.8% of all deaths and 2.2% of all disability-adjusted life years (DALYs) in 2013 [8].

Due to the growing burden of NCDs [3, 4], Ethiopia has developed a comprehensive strategic action plan for the prevention and control of NCDs and associated risk factors focusing on a reduction of risky behaviours including risky dietary habits [9]. Whilst the strategic plan is helpful in guiding interventions, there is a need for a better understanding of the burden of dietary risk factors and their contribution to NCDs in order to achieve the goals of the strategic plan effectively and efficiently. However, there are no adequate national level data or surveillance systems to identify risk factors. In particular, to the best of our knowledge, data on diet quality are lacking, and the contribution of diet to the national burden of disease has not been investigated.

For the first time, we systematically assessed diet-related burden of NCDs (deaths, DALYs, years lived with disability (YLDs) and years of life lost (YLLs)) and the trend over the past two decades in Ethiopia using the Global Burden of Disease (GBD) 2013 data and methods [8, 10]. The study will help to understand the current burden of disease associated with dietary risks in the country. It can be also used as a baseline for the NCDs strategic action plan developed in 2015/16 [9].

## Methods

The GBD databases (GBD 2013) were used to undertake the present study [10]. Using GBD 2013, this study provided estimates of chronic disease burden related to dietary risks in Ethiopia, by sex and age, between 1990 and 2013. A detailed description of the GBD 2013 methods for estimating the burden of disease associated with risk factors has been published elsewhere [8]. Below, we provide a summary of the dietary data sources and methods of estimating the burden of disease related to dietary risks.

### Selection of dietary risk factors

The GBD 2013 selected dietary risks based on their significance to the burden of disease, availability of sufficient data, strength of epidemiological evidence on causality and generalizability [8]. To assess the strength of the epidemiological evidence on the causal relationship between each dietary risk factor and disease, the World Cancer Research Fund evidence grading criteria [11] were used. Diet-disease pairs with convincing or probable evidence were included in the study. Overall, 14 dietary risk factors (eight food types, five nutrients and fibre intake) were included in GBD 2013. These included diet low in fruits, vegetables, whole grain, nuts and seeds, milk, fibre, calcium, seafood omega-3, polyunsaturated fatty acids, and diet high in red and processed meat, sugar-sweetened beverages, *trans* fatty acids, and sodium [8]. Details of these dietary risks are given in Additional file 1: Table S1.

### Dietary exposure levels and data sources

DisMod-MR 2.0, a Bayesian hierarchical meta-regression method, was used to estimate the exposure levels of each dietary risk factor by age, sex, and year. In the analysis, four levels (hierarchies) (global, super-region, region and country) were used. DisMod-MR 2.0 uses tabulated dietary data with uncertainty measurements and has two components. The first component is a mixed effect meta-regression analysis, using sex and covariates as fixed effects by super-region, region, and country. The second component is a cascade repeating the above model by limiting data to 1 year-sex and a hierarchy (for instance, country). This algorithm allows the most use of local data to inform country estimates while taking advantage of sharing data at different levels. Therefore, in the absence of data specific to Ethiopia, we used exposure data from region, super-region or global levels and adjusting for country-level covariates [8].

For each dietary risk factor, a systematic review of the literature was conducted to identify nationally or sub-nationally representative dietary or household budget surveys. For trans-fatty acids, industry data on availability of partially hydrogenated vegetable oil were used. The definition of dietary risk factors were standardized across the surveys. Exposure level of each risk factor was adjusted for energy intake (2000 Kcal/day) using a residual method. For sodium, urinary sodium was used as the main exposure. Data on dietary sodium were converted to urinary equivalent using a multiplier estimated from the surveys reporting both dietary and urinary sodium. Industry data of trans-fatty acids (of partially hydrogenated vegetable oil) were adjusted for the level of gold-standard dietary survey data [8].

Data from the World Health Organization (WHO) (STEPS NCD risk factors survey in Addis Ababa) [12] and the United Nations (Food and Agriculture Organization (FAO) food balance sheets) [13] for Ethiopia were used [8]. Particularly, for six dietary factors (vegetables, whole grains, nuts and seeds, red meat, milk, and seafood omega-3 fatty acids), FAO food balance sheets were used [13]. Individual level data only on vegetable and fruit consumption were available in the STEPS NCD risk factors survey conducted in Ethiopia. Therefore, models for exposure levels of vegetables and fruits were mostly informed by these data. For all other dietary risk factors, we used data from the region (Eastern sub-Sahara Africa), super-region (sub-Sahara Africa) or global level estimates by adjusting for country level covariates. A data representativeness index (DRI), the fraction of countries for which we have identified any data on the risk factor exposure, was computed. The DRI ranged from 17% for polyunsaturated fatty acids to 94% for vegetables [8] (Additional file 1: Table S1).

### Estimating effect sizes

For each diet-disease pair, the relative risk of disease burden was either obtained from new meta-analyses or from previous meta-analyses of prospective observational studies and randomized controlled trials conducted anywhere across the globe. The same relative risks of dietary risk factors were used across countries, including Ethiopia, for a specific sex-age group. Detailed methods and all the data sources included to obtain the relative risks of dietary risks and disease outcomes can be found elsewhere [8]. For diet-disease pairs where evidence is only available on either mortality or morbidity, estimated relative risks were applied equally to both. Due to a lack of high quality studies or very sparse (or unestablished) evidence of a direct link, the effect of sodium and sugar-sweetened beverages on disease outcomes was determined using a two-stage indirect approach. We chose systolic blood pressure and body mass index to estimate the relative risks of high sodium intake and sugar-sweetened beverages, respectively, as the available evidence is of high quality and adequate. To estimate the impact of high sodium intake on disease outcome, first, the relationship between 24-h sodium excretion and change in systolic blood pressure was determined. Then, the link between change in blood pressure and disease outcomes was quantified to estimate the impact of sodium on outcomes [8, 14]. The same approach was used to determine the relative risk of sugar-sweetened beverages using body mass index [8, 15].

### Estimating the effect of diet on disease burden and uncertainties

Methods of GBD to estimate the disease burden (fatal and non-fatal) associated with all risk factors are described elsewhere [2, 3, 16]. For each dietary risk factor, we quantified the proportion of disease burden that could have been prevented if the exposure level had been sustained at the level associated with the lowest risk. This level of exposure, defined as the theoretical minimum risk exposure level (TMREL), determines a single exposure level that reduces risk of all diseases. TMRELs were created using optimal intake from the literature and national recommendations of dietary item consumption. A 20% of uncertainty below and above the mean optimal intake was used to construct the uncertainty intervals of TMRELs. The main inputs to the analyses of the proportion of disease burden attributed to dietary risks were: 1) the exposure level for each dietary risk factor (p); 2) the effect size (RR); 3) the TMREL; and 4) the total number of burden of disease from specific disease mortality and morbidity. Using the first three parameters, the population attributable fraction (PAF) for each diet-disease pair by age (a), sex (s), and year (t) was estimated. Then, disease-specific (o) PAFs and burden of disease were used to calculate the total attributable burden of disease (w). The general equation is provided as follow [8]:

$$PA{F}_{ast} = \frac{{\displaystyle {\int}_{x=l}^u}R{R}_{as}(x){P}_{ast}(x)dx - R{R}_{as}(TMREL)}{{\displaystyle {\int}_{x=l}^u}R{R}_{as}(x){P}_{ast}(x)dx}$$

$$Total\ attributable\ burde{n}_{ast}={\displaystyle \sum_{o=1}^w} Burde{n}_{oast}PA{F}_{oast}$$

The uncertainty of parameters (exposure, relative risk and attributable burden of disease) were estimated using the Monte Carlo approach. All calculations were repeated 1000 times using one draw of each parameter at each iteration. Using the final 1000 draws, the mean and uncertainty intervals were calculated for the final estimates [8].

## Results

### Burden of disease associated with dietary risks in 2013

Dietary risks of chronic diseases were responsible for 60,402 deaths (95% UI: 44,943-74,898), 8.1% and 23.0% of all and NCD deaths, respectively. The number of DALYs caused by poor diet quality was 1,353,407 (95% UI: 1,010,433-1,672,828) DALYs (3.0 and 9.8% of DALYs of all causes and NCDs, respectively). Moreover, 1,291,703 (95% UI: 961,915-1,599,985) YLLs were diet-related which constituted 17.0% of the YLLs caused by NCDs (Table 1).

**Table 1:** Unstandardized and age-standardized burden of disease related to dietary risks and percentage change between 1990 and 2013 in Ethiopia

Burden metrics	Number (95% UI)			Rate (per 100,000) (95% UI)			Proportion (%) (95% UI)		
	1990	2013	Change (%)	1990	2013	Change (%)	1990	2013	Change (%)
Unstandardized (all causes)									
Deaths	37,465 (28,600–47,254)	60,402 (44,943–74,898)	61.2%	78 (60–99)	64 (48–80)	-17.9%	4.2% (3.2–5.3)	8.1% (6.2–10.0)	91.0%
DALYs	95,3087 (717,432–1,208,306)	1,353,407 (1,010,433–1,672,828)	42.0%	1989 (1497–522)	1439 (1074–1779)	-27.7%	1.6% (1.2–2.0)	3.0% (2.3–3.7)	87.8%
YLLs	915,402 (686,597–1,159,486)	1,291,703 (961,915–1,599,985)	41.1%	1910 (1433–2420)	1373 (1023–1701)	-28.1%	1.7% (1.3–2.1)	3.5% (2.7–4.4)	112.1%
YLDs	37,685 (23,070–57,337)	61,704 (37,660–91,841)	63.7%	79 (48–120)	66 (40–98)	-16.6%	0.08% (0.05–0.11)	0.01% (0.01–0.01)	-87.5%
Age-standardized (all causes)									
Deaths	-	-	-	217 (164–274)	183 (135–226)	-16.0%	9.2% (7.0–11.4)	12.8% (9.8–15.7)	40.3%
DALYs	-	-	-	4597 (3503–5815)	3472 (2590–4290)	-24.5%	4.5% (3.4–5.7)	6.4% (4.8–7.9)	42.0%
YLLs	-	-	-	4411 (3369–5560)	3322 (2472–4104)	-24.7%	5.0% (3.8–6.2)	7.9% (6.1–9.7)	58.4%
YLDs	-	-	-	186 (114–280)	150 (90–225)	-19.3%	1.4% (0.9–1.9)	1.2% (0.9–1.7)	-10.1%
Unstandardized (NCD burden)									
Deaths	-	-	-	-	-	-	21.0% (16.0–26.8)	23.0% (17.9–27.9)	9.5%
DALYs	-	-	-	-	-	-	10.1% (7.3–13.3)	9.8% (7.4–12.4)	-3.0%
YLLs	-	-	-	-	-	-	14.3% (9.9–19.6)	17.0% (13.3–21.0)	18.9%
YLDs	-	-	-	-	-	-	1.2% (0.9–1.7)	1.0% (0.7–1.4)	-16.7%
Age-standardized (NCD burden)									
Deaths	-	-	-	-	-	-	24.8% (19.3–31.1)	25.3% (19.5–30.7)	2.0%
DALYs	-	-	-	-	-	-	15.6% (12.0–19.7)	14.7% (11.2–18.2)	-5.8%
YLLs	-	-	-	-	-	-	21.8% (16.9–27.2)	23.0% (17.9–27.7)	5.5%
YLDs	-	-	-	-	-	-	2.0% (1.4–2.8)	1.6% (1.2–2.3)	-20.0%

*DALYs* disability-adjusted life years; *YLLs* years of life lost; *YLDs* years lived with disabilities; *NCD* non-communicable disease; *UI* uncertainty interval

**Fig. 1:**
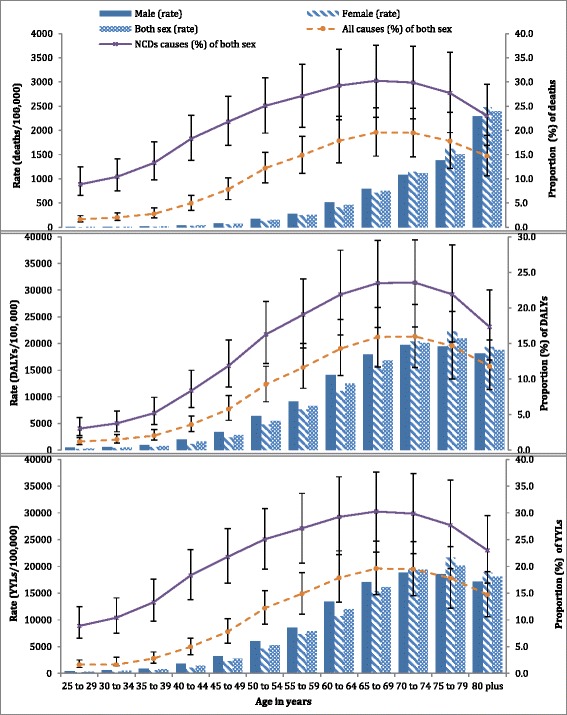
Diet-related burden of disease (deaths, disability-adjusted life years (DALYs) and years of life lost (YLLs)) and the proportion of contribution to the burden of all causes and non-communicable diseases (NCDs) by age and sex in Ethiopia for 2013

Males had slightly higher diet-related burden of disease, although it was not significantly different to females. It was also found that the diet-related mortality rate was highest among those aged 50 to 69 years, where 28.5% of all NCD deaths were related to poor diet. Above the age of 80 years, 2393 deaths per 100,000 were associated with dietary risks. In this age group, the proportion of diet-related NCD mortality was 23.0% which is lower than people in the 65–69 years age group. Diet-related DALYs had a similar pattern as mortality, peaking between the age of 75 and 79 (20,912 DALYs/100,000). The highest proportion of diet-related NCD DALYs were found among those aged 70-74 years (23.6%) (Fig. 1 and Additional file 1: Figures S1 and S2).

**Fig. 2:**
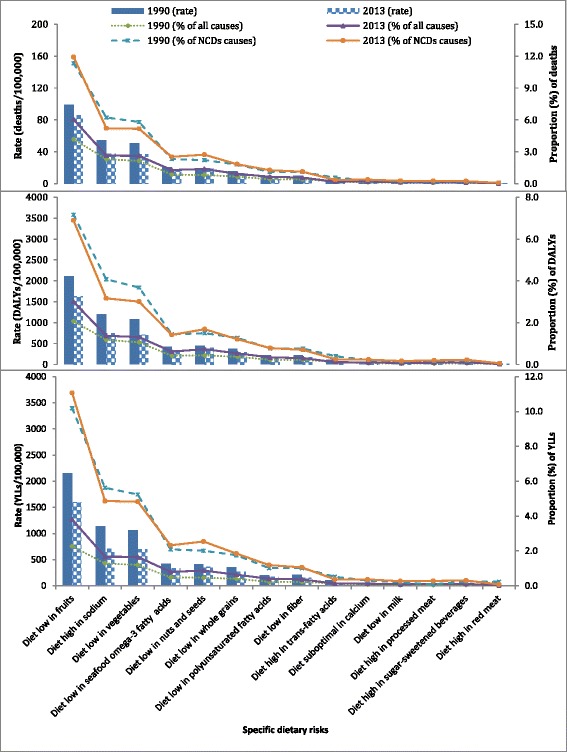
Age-standardized burden of disease (deaths, disability-adjusted life years (DALYs) and year of life lost (YLLs)) related to specific dietary risks and the proportion of contribution to the burden of all causes and non-communicable diseases (NCDs) for 1990 and 2013 in Ethiopia (*The sum of percentages exceeds the total for all dietary risk factors combined because of overlaps between various risk factors.)*

Diets low in fruits, vegetables, nuts and seeds and seafood omega-3 fatty acids, and high in sodium were the top five most important risks associated with NCDs. A diet low in fruits was responsible for 86 deaths per 100,000 or 11.0% of NCD deaths. In the 50–69 years age group, 14.3, 6.1 and 6.3% of NCD deaths were associated with low fruit, vegetable, and high sodium consumption, respectively. Low consumption of fruits accounted for 1632 DALYs per 100,000. Compared to other dietary risks, high intakes of red and processed meat and sugar-sweetened beverages were found to have a lower burden (Fig. 2 and Additional file 1: Figures S2).

Almost half (44.0%), of CVD deaths were predisposed by poor diet quality. Among those aged 50–69 years, 51.7% (23,087 deaths) of CVD deaths were associated with dietary risks. Almost nine of every 10 diet-related deaths (88.4%) were caused by CVD. The total number CVD deaths associated with dietary risks was 53,375, and this constituted 20.4% of all the NCD deaths. Overall, 10.0% of all cancer deaths were related to dietary risks (Table 2).

**Table 2:** Diet-related deaths of specific causes and percentage change by age between 1990 and 2013 in Ethiopia

Age category	Metric	1990 (95% UI)			2013 (95% UI)			Percentage change		
		Diabetes, urogenital, blood, and endocrine diseases	CVD	Cancer	Diabetes, urogenital, blood, and endocrine diseases	CVD	Cancer	Diabetes, urogenital, blood, and endocrine diseases	CVD	Cancer
15 to 49 years	Number of deaths	347 (253–460)	3898 (2730–5232)	486 (284–722)	417 (304–547)	3878 (2772–5140)	603 (366–889)	20.2%	-0.5%	24.1%
	Rate (per 100,000)	1.7 (1.2–2.2)	19 (13–25)	2.3 (1.4–3.4)	0.94 (0.68–1.23)	9 (6–12)	1.4 (0.82–1.99)	-44.7%	-52.6%	-39.1%
	Proportion (%) of all deaths	0.2%	2.5%	0.3%	0.3%	2.4%	0.4%	13.0%	-5.1%	6.3%
	Proportion (%) within the specific disease death	8.8% (6.8–11.4)	38.9% (30.3–50.0)	6.9% (4.3–10.0)	8.5% (6.4–11.0)	37.1% (29.8–46.8)	6.7% (4.2–9.7)	-3.4%	-4.6%	-3.9%
50 to 69 years	Number of deaths	874 (634–1150)	16,466 (12,326–21,122)	1651 (910–2516)	1271 (961–1612)	23,087 (16,911–29,471)	2232 (1278–3392)	45.4%	40.2%	35.2%
	Rate (per 100,000)	22 (16–29)	413 (309–530)	42 (23–63)	17 (13–22)	309 (226–394)	30 (17–45)	-22.7%	-25.2%	-28.6%
	Proportion (%) of all deaths	0.6%	11.6%	1.2%	0.8%	14.5%	1.4%	29.0%	24.8%	20.7%
	Proportion (%) within the specific disease death	13.5% (10.6–16.9)	54.1% (42.3–67.7)	11.3% (6.6–16.8)	14.4% (11.6–17.5)	51.7% (40.4–63.0)	10.6% (6.4–15.3)	6.7%	-4.4%	-6.2%
70+ years	Number of deaths	451 (310–653)	12,426 (9120–16,276)	867 (556–1263)	870 (623–1160)	26,411 (18,983–33,739)	1635 (1102–2312)	92.9%	112.5%	88.6%
	Rate (per 100,000)	53 (36–76)	1450 (1065–1900)	101 (65–147)	46 (33–61)	1394 (1002–1781)	86 (58–122)	-13.2%	-3.9%	-14.9%
	Proportion (%) of all deaths	0.5%	12.9%	0.9%	0.5%	15.6%	1.0%	8.5%	20.8%	6.7%
	Proportion (%) within the specific disease death	9.4% (6.8–13.4)	44.0% (33.3–57.0)	13.1% (8.9–18.7)	9.4% (6.9–12.4)	40.8% (30.7–50.9)	12.3% (8.5–16.8)	0.0%	-7.3%	-6.1%
Total	Number of deaths	1672 (1271–2135)	32,790 (24,804–41,594)	3004 (1803–4368)	2558 (2056–3164)	53,375 (39,172–66,645)	4470 (2792–6422)	53.0%	62.8%	48.8%
	Unstandardized rate (per 100,000)	3.5 (2.7–4.5)	68 (52–87)	6 (4–9)	2.7 (2.2–3.4)	57 (42–71)	5 (3–7)	-22.9%	-16.2%	-16.7%
	Age-standardized rate (per 100,000)	9 (7–11)	192 (145–242)	16 (10–24)	7 (6–9)	163 (119–204)	13 (8–18)	-22.2%	-15.1%	-18.8%
	Unstandardized proportion (%) out of all deaths	0.2%	3.7%	0.3%	0.3%	7.1%	0.6%	78.9%	93.0%	76.5%
	Unstandardized proportion (%) out of all NCDs deaths	0.9%	18.3%	1.7%	1.0%	20.4%	1.7%	11.1%	11.5%	0.0%
	Unstandardized proportion (%) within the specific disease death	9.0% (6.9–11.4)	46.1% (36.3–57.0)	10.1% (6.4–14.2)	10.0% (8.1–12.1)	44.0% (34.3–52.8)	9.8% (6.3–13.7)	11.1%	-4.6%	-3.0%
	Age-standardized proportion (%) within the specific disease death	10.2% (8.1–12.9)	45.6% (35.6–56.7)	11.5% (7.6–16.1)	10.6% (8.6–13.0)	42.9% (33.3–52.0)	10.9% (7.1–15.0)	3.9%	-5.9%	-5.2%

*CVD* cardiovascular disease; *NCD* non-communicable disease; *UI* uncertainty interval

### Trend of disease burden related to dietary risks between 1990 and 2013

There was a non-significant decreasing pattern of age-standardized rate of diet-related burden of disease. In 1990, there were 4597 diet-related DALYs per 100,000 which decreased to 3472 DALYs per 100,000 in 2013. In terms of absolute number and proportion of burden, however, the burden of disease associated with dietary risks increased over the past two decades. In 2013 (60,402 deaths), the number of NCD deaths associated with poor diet quality increased by 61.2% from the 1990’s estimate (37,465 deaths). The proportion of diet-related NCD deaths and YLLs increased by 2.0% (from 24.8% in 1990 to 25.3% in 2013) and 5.5% (from 21.8% in 1990 to 23.0% in 2013), respectively (Table 1 and Additional file 1: Figure S3).

Diets low in fruits and vegetables and high in sodium were the top three distinct dietary risks with highest contribution to NCD burden over the past two decades. The age-standardized proportion of NCD deaths related to low fruit consumption slightly increased (11.3 and 11.9% in 1990 and 2013, respectively). In 2013, 1632 DALYs per 100,000 were associated with low consumption of fruits, and this decreased by 22.8% from the 1990 estimate (2113 DALYs per 100,000). Similarly, 2154 and 1600 YLLs per 100,000 were associated with low consumption of fruits in 1990 and 2013, respectively (Additional file 1: Figure S4).

The number of diet-related cancer deaths in the 15–49 years age group increased by 24.1% over the past two decades (from 417 to 603 deaths). Across all age groups, there was also a 93.0% increase (from 3.7 to 7.1%) in the age-standardized proportion of diet-related CVD deaths over the years. In people aged 70 years and over, the number CVD deaths increased by 112.5% (from 12,426 to 26,411 deaths). In this age group, the number of diabetes, urogenital, blood, and endocrine, and cancer deaths related to dietary risks also increased by 92.9% (from 451 to 870 deaths) and 88.6% (from 867 to 1635 deaths), respectively (Table 2).

## Discussion

This study systematically assessed the diet-related national burden of disease in Ethiopia using the GBD 2013 data. In 2013, dietary risks of chronic diseases contributed to 60,402 deaths. This constituted 8.1% of all deaths and 23.0% of deaths from the NCDs. The rate of burden of disease associated with poor diet quality has slightly decreased over the past two decades; however, the relative contribution to the NCD burden remained stable. Diet-related burden of disease was common in males and people aged 45 years and above. Diets low in fruits and vegetables and high in sodium were found to be the most common specific dietary risks. The relative contribution of the leading dietary factors to the national burden of disease has slightly increased between 1990 and 2013. In 2013, almost half of CVD deaths were diet-related, and nine of every ten diet-related deaths (88.4%) were due to CVD.

High burden of diet-related NCDs in this study is in line with the existing evidence which demonstrates unhealthy dietary patterns and associated diseases are increasing in low-income countries [17, 18]. The finding is also consistent with the previous reports in other developing countries [19, 20]. Compared to some other sub-Saharan countries, the proportion of diet-related deaths in Ethiopia was high. In Kenya and Uganda, for instance, 18.0 and 21.1% of NCD deaths were related to dietary risks in 2013, respectively [10], compared to 25.3% of Ethiopia. Although the difference is statistically non-significant, we also found that the burden of diet-related disease was higher in males and younger adults. This could be due to the fact that males and younger adults are more likely to consume a low quality diet [17, 21] which consequently results in increased risk of NCD acquisition.

In 2013, behavioural risk factors were responsible for 31.8% (237,472 deaths) of all deaths, 28.8% (13,141,325 DALYs) of all DALYs, and 15.5% (1,355,316 YLDs) of all YLDs in Ethiopia [4, 8], with dietary risks in particular, associated with a quarter (25.4%) of all behavioural related risk factor deaths. Dietary risks were the second (behind undernutrition) and fifth (behind undernutrition, high blood pressure, air pollution, water, sanitation and hygiene) highest ranked risk factor among behavioural related and all risk factors of deaths, respectively [4]. The coexistence of the two nutrition related risks (dietary risks of NCDs and undernutrition), as major contributors to the disease burden, shows the complexity of nutrition transition in the country. In line with this, available evidence shows that the current change in dietary behaviour is faster [22] and has an unusual pattern [18] in developing countries compared to what has been observed in developed nations. Further studies to explicitly explore the impact of this paradox should be considered in Ethiopia.

Dietary risk was the second most common risk factor of all risks of deaths and DALYs in people aged 50 years and over (behind high systolic blood pressure). However, of all behavioural risks, dietary factors were found to be the leading risks in all metrics (mortality, DALYs and YLDs) of burden of disease in this age group [4]. Studies have also shown that dietary behaviours are the major contributors for the burden of NCDs in Africa [18, 23]. Factors such as limited availability and accessibility of nutrient-dense foods and increased consumption of processed foods could be the driving forces [24]. The other possible reasons could be the economic development and trade related policies in the country which may result in an increased consumption of diet with poor quality [18, 24]. Although there is lack of evidence on the impact of economic development and trade policy on the overall food consumption pattern in Ethiopia, the expenditure on processed foods and per capita energy consumption increased between 1996 and 2011 as a result of raised household income [25]. Over the same period, expenditure on some of the nutrient-dense foods (including pulses and unprocessed cereals) decreased [25], and the consumption of animal products remained consistent [26].

Low intakes of fruits and vegetables caused the highest burden of disease compared to other dietary risks. Epidemiological evidence shows that low intakes of fruits and vegetables are associated with multi-morbidity and overall mortality [27, 28]. In South Africa, low consumption of fruits and vegetables accounted for 3.2% of all deaths and 1.1% of all DALYs [28]. To date, the magnitude of NCD burden associated with low intakes of fruits and vegetables remains unstudied in Ethiopia. Although the consumption of fruits and vegetables has increased between 1996 (31 kg/adult equivalent/year) and 2011 (45 kg/adult equivalent/year) [29], the amount is still far below the recommended level (a minimum of 400 g of fruits and vegetables per day). In line with this, studies have shown that the cultivation and consumption of fruits and vegetables are very low in the country [30–32]. A study in an urban area (Gondar town) also indicated that consumption of vegetables (87.3% consumed only 0–3 days per week) and fruits (96.8% consumed only 0–3 times per week) was very low [33]. According to the 2006 WHO report, 98.9% of the population consumed less than five servings of fruits and vegetables per day in the capital (Addis Ababa) [12]. Several reasons, such as social and cultural preferences for animal products, lack of consumer awareness, economic constraints, absence of nutrition intervention programme [32] could contribute to the low consumption of fruits and vegetables in the country. In addition, lack of awareness of the storage and preparation, when available, could also be another factor for the low consumption [34].

In the current study, consumption of a diet high in sodium was also one of the leading dietary risk factors, contributing to 6.3% of NCD deaths. A high intake of sodium is associated with increased blood pressure eventually resulting in CVD [35]. It is also linked with increased mortality risks from all causes [36]. Of the 1.65 million global deaths from CVD causes and attributed to high sodium intake, 84.3% were in low- and middle-income countries [14]. In Mauritius, for instance, the proportion of cardiovascular mortality associated with high consumption of sodium was 27.4% [14]. Morbidity and mortality due to CVD are prevalent in Ethiopia [5, 33]. Although the mean sodium intake is higher (2.27 g/day) [37] than the WHO recommended limit (2 g/day), the associated burden of NCDs has not been investigated in previous studies. In addition, despite the fact that public health interventions against high consumption of sodium are effective in reducing associated burden of disease [38, 39], these interventions are not available in Ethiopia.

It has been demonstrated that public health interventions targeting risk factors of NCDs, including dietary risks, are cost effective [38, 40]. Particularly, evidence shows that addressing dietary risks through nutrition policies and multi-sectoral collaboration are cost effective in mitigating burden of NCDs in developing countries [41, 42]; however, efforts are very limited [20]. In Ethiopia, emphasis has been given to interventions for infectious diseases and maternal health and child undernutrition [43, 44] although the burden of NCDs is high [5, 45]. However, in recent years the government of Ethiopia has acknowledged the increasing burden of NCDs and hence developed a prevention and control strategic action plan [9]. Nevertheless, the implementation of this plan could face challenges including lack of national level data on the relative contribution of specific risk factors to the burden NCDs which would help targeted interventions.

Dietary data are extremely limited in Ethiopia. As a result, most of our models used data from region, super-region or global level estimates. In spite of this, the findings of this study clearly highlight the urgent need for the formulation of a nutrition policy and dietary guidelines for the general population. In this regard, the contribution of this study will be two-fold. Firstly, it can be used as a baseline and input for the recent interventions on NCD risk factors. Secondly, it can serve as an evidence for the development of food policies, programs and dietary guidelines in Ethiopia. This study further underlines the necessity of initiatives to systematically collect and organize important local dietary data using appropriate and up-to-date methods. Researchers should also explore dietary risk factors for NCDs using primary data. Explicit assessment of the root factors of dietary behaviours which are specific to the country should be conducted at multiple (individual, sociocultural, community, agricultural, government and global factors) levels in order to design effective and tailored interventions and strategies.

Although this paper is based on the GBD 2013 study [8] which used robust methods to organize and analyse data, potential limitations should be considered in the interpretation of the findings. First, except for vegetable and fruit, we estimated dietary risk exposure levels from region, super-region or global level data. Although we used country-level covariates to adjust these data, it is important to note that the statistical uncertainty of estimates could increase. Even for diets low in vegetables and fruits, only a survey in Addis Ababa (the capital of Ethiopia) [12] was used which could underplay the representativeness for other regions of the country, particularly the rural areas. Given each of these limitations, the possibility of both overestimation and underestimation cannot be ruled out and the wide uncertainty intervals of the burden of disease estimates are evident. In this regard, we included the uncertainties in the analysis of burden of disease estimates [8].

Secondly, the effect of diets high in sodium and sugar-sweetened beverages on disease outcomes was assessed using a different approach (through systolic blood pressure and body mass index, respectively) [8], which may impact comparability with the other dietary risk factors. In addition, the risk factors could have different pathways other than these (e.g. sugar-sweetened beverages could have an effect through change in blood glucose level [46], although the evidence is not as strong as body mass index). Thirdly, in estimating the relative risks, potential confounders and mediators could affect the relationship between the dietary risk factors and disease outcomes. In addition, the correlation among the dietary risk factors could be another potential limitation. For instance, people who eat more fruits are more likely to consume more vegetables, affecting estimates of relationship with disease outcomes. The possibility of residual confounding cannot be excluded, although we used relative risks from meta-analyses of the observational studies that had considered the major confounders. Fourthly, use of universal effect size (relative risks) across countries for a given age-sex group is considered a drawback of this study because dietary risks could have different effect on disease outcomes across different population subgroups [8].

## Conclusions

In summary, the burden of disease associated with a poor quality diet is high. Despite the fact that most people eat plant based foods [25, 47, 48], diets low in fruits and vegetables are found to be the most common dietary risk factors contributing to a large portion of diet-related NCD burden. Diets high in sodium and low in seafood and omega-3 fatty acids were also common. The findings of this study underline the importance of designing and implementing nutrition policies, programs and dietary guidelines that can effectively address dietary risks. To mitigate the impact of oncoming NCDs in Ethiopia, multisectoral approaches, mainly involving the agricultural and health sector, are required. At a community level, creating synergistic efforts by coordinating the activities of the health and agricultural extension workers is essential.

Coordinated efforts and interventions should focus on improving consumption of vegetables and fruits. Designing an intervention aimed towards a targeted restriction of salt consumption, taking into account the current universal salt iodization program in the country, should be also considered. Increasing awareness of the community on the importance of a quality diet, and increasing availability and accessibility of diets rich in important nutrients, such as seafood, may help in the prevention of NCD burden in the country. NCDs and risk factor surveillance systems, using improved data collection technologies and focusing on behavioural factors (including the dietary risk factors), should be designed and implemented.

## Additional file

Additional file 1: Table S1. Dietary Risk factors, definitions, and Minimum theoretical risk exposure levels and data representative index *(Source: GBD 2013Risk factors study).* **Figure S1.** Burden of disease (deaths, disability-adjusted life years (DALYs) and years of life lost YLLs)) with 95% uncertainty interval related to dietary risks in Ethiopia for 1990 and 2013. **Figure S2.** The proportions of non-communicable diseases burden related to the top five dietary risks by age category in Ethiopia for 2013 (*The sum of percentages in rows exceeds the total for all dietary risk factors combined because of overlaps between various risk factors.).* **Figure S3.** Age-standardized diet-related burden of disease (deaths, disability-adjusted life years (DALYs) and year of life lost (YLLs)) with 95% uncertainty interval and the proportion of contribution to the burden of all cause and non-communicable diseases (NCDs) by sex between 1990 and 2013 in Ethiopia. **Figure S4.** Age-standardized diet-related proportion of non-communicable diseases burden between 1990 and 2013 in Ethiopia (*The sum of percentages in rows exceeds the total for all dietary risk factors combined because of overlaps between various risk factors.). (PDF 430 kb)*

## Abbreviations

CVD: Cardiovascular disease
DALYs: Disability-adjusted life years
FAO: Food and Agriculture Organization
GBD: Global Burden of Disease
IHME: Institute of Health Metrics and Evaluation
NCD: Non-communicable disease
TMREL: Theoretical minimum risk exposure levels
WHO: World Health Organization
YLDs: Years lost due to disability
YLLs: Years of life lost
