# Supplementary material for: The impact of dietary risk factors on the burden of non-communicable diseases in Ethiopia: findings from the Global Burden of Disease study 2013
Source: Int J Behav Nutr Phys Act. 2016 Dec 16;13:122. doi: 10.1186/s12966-016-0447-x (PMC5159959; doi:10.1186/s12966-016-0447-x)
Supplement: Additional file 1: Table S1. — Dietary Risk factors, definitions, and Minimum theoretical risk exposure levels and data representative index (Source: GBD 2013Risk factors study). Figure S1. Burden of disease (deaths, disability-adjusted life years (DALYs) and years of life lost YLLs)) with 95% uncertainty interval related to dietary risks in Ethiopia for 1990 and 2013. Figure S2. The proportions of non-communicable diseases burden related to the top five dietary risks by age category in Ethiopia for 2013 (The sum of percentages in rows exceeds the total for all dietary risk factors combined because of overlaps between various risk factors.). Figure S3. Age-standardized diet-related burden of disease (deaths, disability-adjusted life years (DALYs) and year of life lost (YLLs)) with 95% uncertainty interval and the proportion of contribution to the burden of all cause and non-communicable diseases (NCDs) by sex between 1990 and 2013 in Ethiopia. Figure S4. Age-standardized diet-related proportion of non-communicable diseases burden between 1990 and 2013 in Ethiopia (The sum of percentages in rows exceeds the total for all dietary risk factors combined because of overlaps between various risk factors.). (PDF 430 kb) [file 12966_2016_447_MOESM1_ESM.pdf]

Supplementary table 1: Dietary Risk factors, definitions, and Minimum theoretical risk exposure levels and data representative index *(**Source**: GBD 2013Risk factors study)*

**Risk factors**	**Definition**	**Theoretical minimum risk exposure level**	**DRI**
Diet low in fruits	Average daily consumption of fruits (fresh, frozen, cooked, canned, or dried, excluding fruit juices and salted or pickled fruits)	Consumption of fruit between 200 g and 400 g per day	56.4%
Diet low in vegetables	Average daily consumption of vegetables (fresh, frozen, cooked, canned, or dried vegetables, including legumes but excluding salted or pickled vegetables, juices, nuts and seeds, and starchy vegetables such as potatoes or corn)	Consumption of vegetables between 350 g and 450 g per day	93.6%
Diet low in whole grains	Average daily consumption of whole grains (bran, germ, and endosperm in their natural proportion) from breakfast cereals, bread, rice, pasta, biscuits, muffins, tortillas, pancakes, and other sources	Consumption of whole grains between 100 g and 150 g per day	89.9%
Diet low in nuts and seeds	Average daily consumption of nut and seed foods	Consumption of nuts and seeds between 12 g and 20 g per day	86.7%
Diet low in milk	Average daily consumption of milk, including non-fat, low fat, and full-fat milk, excluding soy milk and other plant derivatives	Consumption of milk between 425 g and 475 g per day	91.0%
Diet high in red meat	Average daily consumption of red meat (beef, pork, lamb, and, goat but excluding poultry, fish, eggs, and all processed meats)	Consumption of red meat between 11・4 g and 17・1 g per day	91.0%
Diet high in processed meat	Average daily consumption of meat preserved by smoking, curing, salting, or addition of chemical preservatives	Consumption of processed meat between 0 g and 14・3 g per day	28.2%
Diet high in sugar sweetened beverages	Average daily consumption of beverages with ≥50 kcal per 226・8 g serving, including carbonated beverages, sodas, energy drinks, and fruit drinks, but excluding 100% fruit and vegetable juices	Consumption of sugar-sweetened beverages between 0 g and 64・3 g per day	27.1%
Diet low in fibre	Average daily intake of fibre from all sources including fruits, vegetables, grains, legumes, and pulses	Consumption of fibre between 28 g and 32 g per day	27.1%
Diet suboptimal in calcium	Average daily intake of calcium from all sources, including milk, yogurt, and cheese	Consumption of calcium between 0 g and 0・77 g per day	31.4%
Diet low in seafood omega-3 fatty acids	Average daily intake of eicosapentaenoic acid and docosahexaenoic acid	Consumption of seafood omega-3 fatty acids between 200 mg and 300 mg per day	90.4%
Diet low in polyunsaturated fatty acids	Average daily intake of omega-6 fatty acids from all sources, mainly liquid vegetable oils, including soybean oil, corn oil, and safflower oil	Consumption of polyunsaturated fatty acids between 10% and 15% of total daily energy	17.0%
Diet high in trans fatty acids	Average daily intake of trans fat from all sources, mainly partially hydrogenated vegetable oils and ruminant products	Consumption of trans fatty acids between 0% and 0・8% of total daily energy	42.0%
Diet high in sodium	24 h urinary sodium measured in mg per day	Consumption of sodium between 1 g and 5 g per day	33.5%

Data representativeness index (DRI): *The DRI for a risk factor is the fraction of countries for which we have identified any data for the risk factor between 1985 and 2013.*

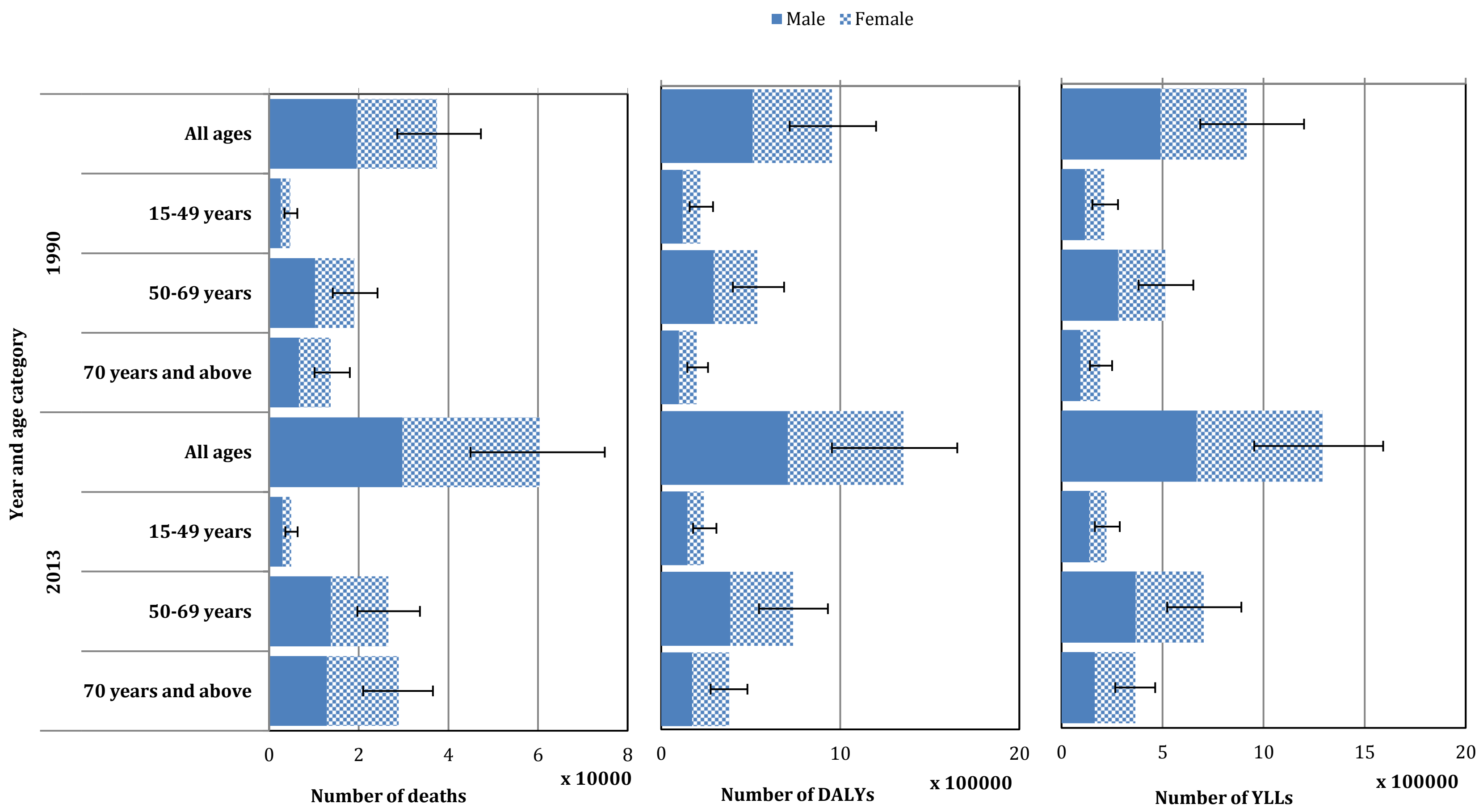

Supplementary Figure 1: Burden of disease (deaths, disability-adjusted life years (DALYs) and years of life lost YLLs)) with 95% uncertainty interval related to dietary risks in Ethiopia for 1990 and 2013

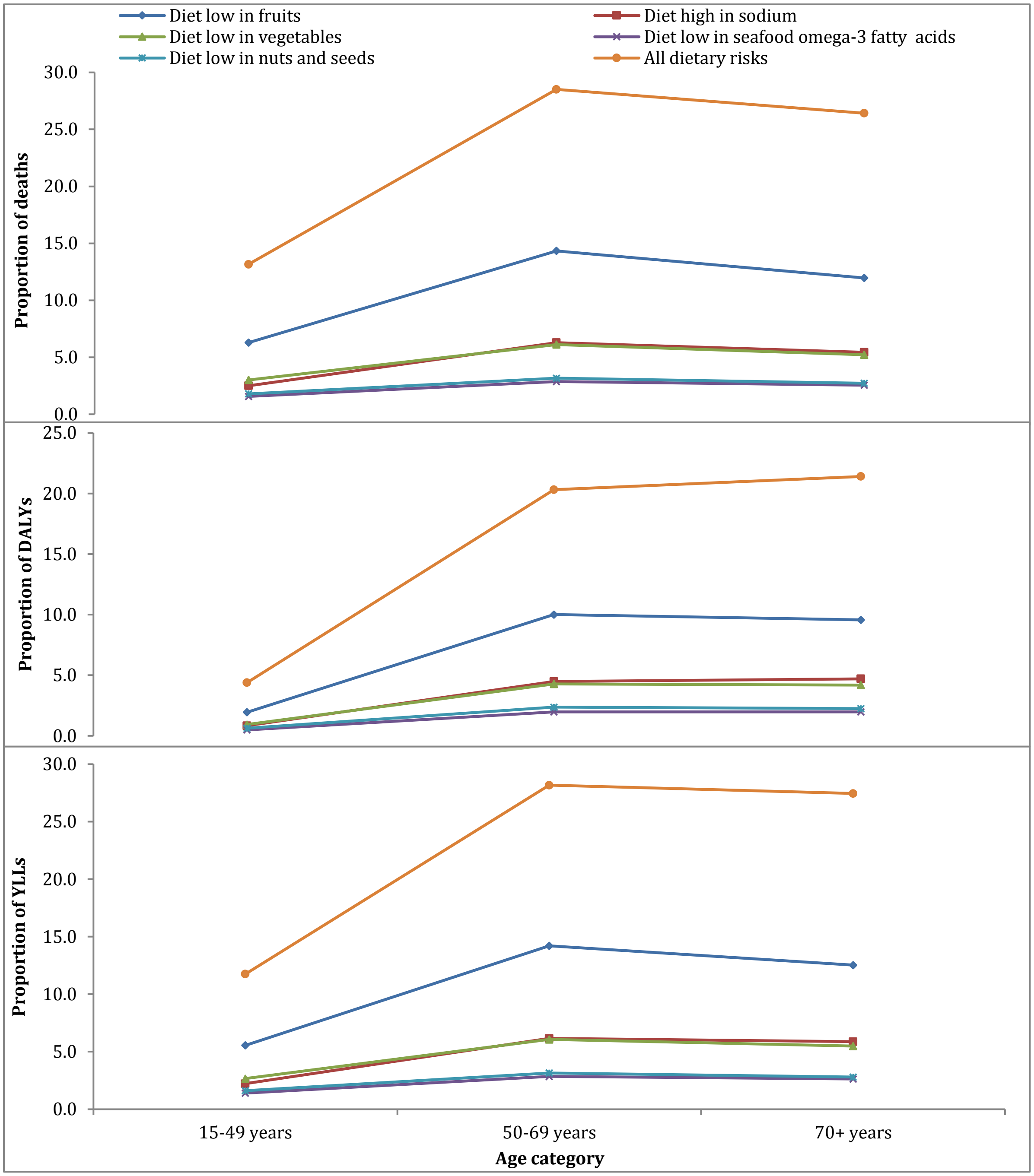

Supplementary Figure 2: The proportions of non-communicable diseases burden related to the top five dietary risks by age category in Ethiopia for 2013 (*The sum of percentages in rows exceeds the total for all dietary risk factors combined because of overlaps between various risk factors.)*

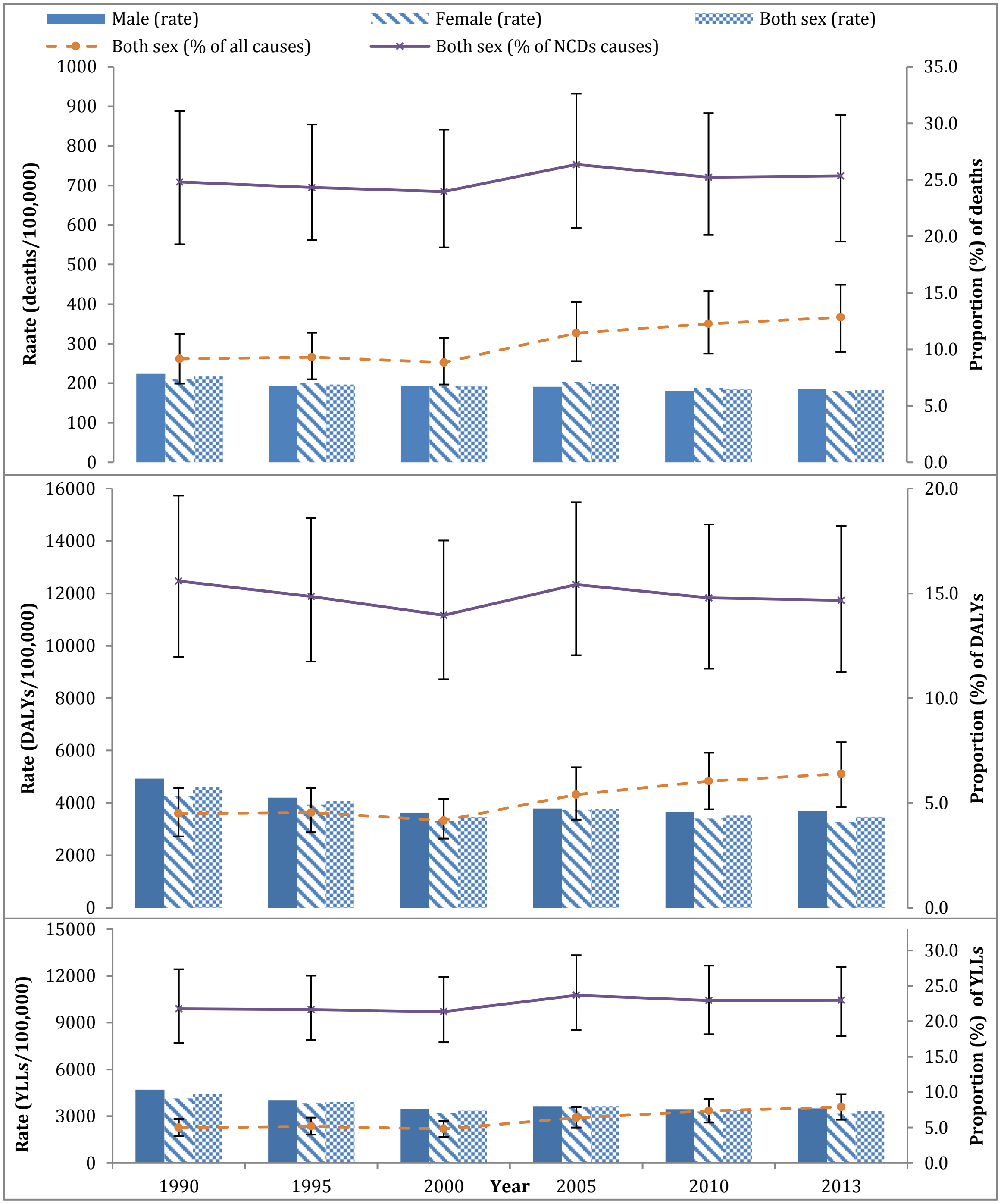

Supplementary Figure 3: Age-standardized diet-related burden of disease (deaths, disability-adjusted life years (DALYs) and year of life lost (YLLs)) with 95% uncertainty interval and the proportion of contribution to the burden of all cause and non-communicable diseases (NCDs) by sex between 1990 and 2013 in Ethiopia

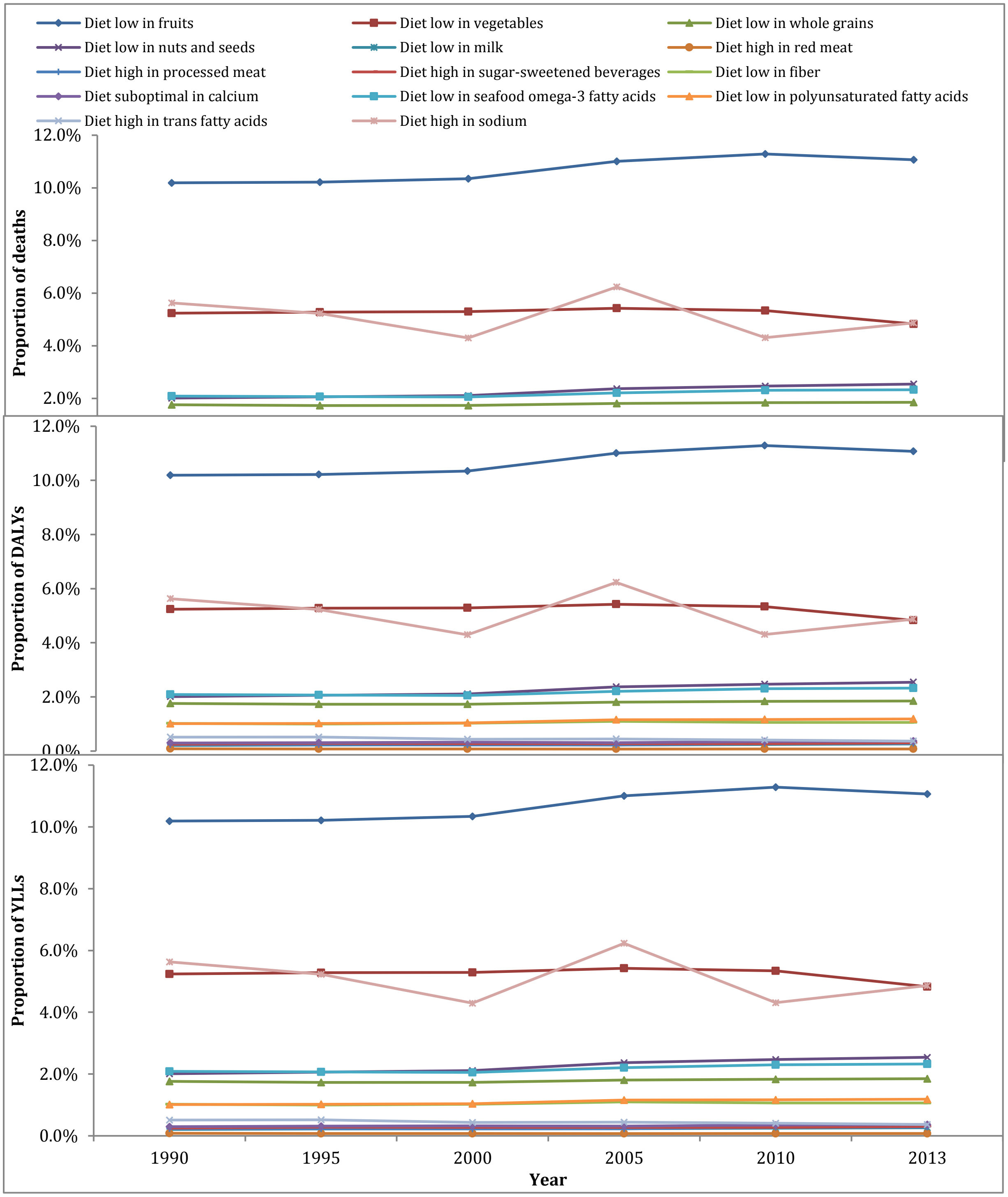

Supplementary Figure 4: Diet-related proportion of non-communicable diseases burden between 1990 and 2013 in Ethiopia (*The sum of percentages in rows exceeds the total for all dietary risk factors combined because of overlaps between various risk factors.)*
